# The Two Sides of Sensory–Cognitive Interactions: Effects of Age, Hearing Acuity, and Working Memory Span on Sentence Comprehension

**DOI:** 10.3389/fpsyg.2016.00236

**Published:** 2016-02-29

**Authors:** Renee DeCaro, Jonathan E. Peelle, Murray Grossman, Arthur Wingfield

**Affiliations:** ^1^Department of Psychology and Volen National Center for Complex Systems, Brandeis University, WalthamMA, USA; ^2^Department of Otolaryngology, Washington University in St. Louis, St. LouisMO, USA; ^3^Department of Neurology, University of Pennsylvania, PhiladelphiaPA, USA

**Keywords:** working memory, hearing acuity, sentence comprehension, adult aging, syntactic structure

## Abstract

Reduced hearing acuity is among the most prevalent of chronic medical conditions among older adults. An experiment is reported in which comprehension of spoken sentences was tested for older adults with good hearing acuity or with a mild-to-moderate hearing loss, and young adults with age-normal hearing. Comprehension was measured by participants’ ability to determine the agent of an action in sentences that expressed this relation with a syntactically less complex subject-relative construction or a syntactically more complex object-relative construction. Agency determination was further challenged by inserting a prepositional phrase into sentences between the person performing an action and the action being performed. As a control, prepositional phrases of equivalent length were also inserted into sentences in a non-disruptive position. Effects on sentence comprehension of age, hearing acuity, prepositional phrase placement and sound level of stimulus presentations appeared only for comprehension of sentences with the more syntactically complex object-relative structures. Working memory as tested by reading span scores accounted for a significant amount of the variance in comprehension accuracy. Once working memory capacity and hearing acuity were taken into account, chronological age among the older adults contributed no further variance to comprehension accuracy. Results are discussed in terms of the positive and negative effects of sensory–cognitive interactions in comprehension of spoken sentences and lend support to a framework in which domain-general executive resources, notably verbal working memory, play a role in both linguistic and perceptual processing.

## Introduction

Unlike reading, where one can control the input rate with eye-movements, in the case of spoken language speech rate is controlled by the speaker and not by the listener. Because of the rapidity of natural speech and its inherently transient nature, comprehension operations that cannot be accomplished as the speech is being heard must be conducted on a fading trace of that speech in memory (Jarvella, 1970, 1971; Fallon et al., 2004). Added to the rapidity of natural speech, many of the words we hear in spoken discourse are significantly under-articulated, requiring a heavy demand on acoustic and linguistic context for successful recognition (Pollack and Pickett, 1963; Lindblom et al., 1992; Wingfield et al., 1994).

Adult aging brings special challenges for speech comprehension due to age-related declines in episodic memory (Wingfield and Kahana, 2002), processing speed (Salthouse, 1996), and working memory resources (Salthouse, 1994), all of which can have a negative impact on comprehension of spoken sentences (see reviews in Light, 1990; Carpenter et al., 1994; Wingfield and Lash, 2016). Of special note, however, is the effect on sentence comprehension of age-related hearing impairment. The goal of this present study is to examine the effects of hearing impairment in older adults on the comprehension of spoken sentences as the processing difficulty is manipulated by the syntactic complexity of the sentences and the sound level of the presented stimuli.

### Hearing Acuity and Sentence Comprehension

Age-related hearing loss is the third most prevalent chronic medical condition among older adults, exceeded only by arthritis and hypertension (Lethbridge-Cejku et al., 2004). This is of concern for speech comprehension as even with a relatively mild hearing loss one can miss, or mishear, words from spoken utterances. More subtle, however, is the mounting evidence that even with a relatively mild hearing loss the cognitive effort needed for successful front-end speech recognition can draw resources that would otherwise be available for storing what has been heard in memory (Rabbitt, 1991; Surprenant, 1999, 2007; Pichora-Fuller, 2003; Wingfield et al., 2005), or comprehending sentences in which the meaning is expressed with complex syntax (Wingfield et al., 2006). Critically, this effect can occur even when it can be demonstrated that the speech itself has passed a threshold of audibility.

When the consequences of this front-end perceptual effort are added to an age-related decline in working memory capacity (e.g., Salthouse, 1994), one might expect speech comprehension to be far poorer among older adults than one ordinarily observes. There is a general recognition in the literature that older adults’ relative success with language comprehension is due to their ability to offset effects of reduced hearing acuity and working memory resources with the compensatory use of linguistic knowledge that is ordinarily well-preserved in healthy aging. (Reviews of evidence for the preservation of linguistic knowledge and the procedural rules for its use in healthy aging can be found in, for example, Light, 1988; Kemper, 1992; Kempler and Zelinski, 1994; Wingfield and Stine-Morrow, 2000.)

This delicate balance between the negative effects of processing deficits and the positive effects of spared linguistic knowledge in adult aging works well until the total processing burden exceeds a listener’s processing capacity. When compensatory mechanisms are not able to keep up with demand, listeners’ performance will suffer. Increasing the processing challenge through linguistic and acoustic manipulations is therefore a useful way to test the interaction of cognitive and perceptual factors in speech comprehension. In the following experiment, we examine spoken sentence comprehension under conditions in which this balance is maintained, and under conditions where the processing challenge disrupts this balance by increasing the processing demands needed for successful comprehension at the linguistic and perceptual levels.

### Syntactic Complexity and Working Memory

In addition to the challenge imposed on many older adults by a reduced quality of the acoustic signal, challenges also arise when the syntactic structure of a sentence departs from a simple canonical form in which the first noun in the sentence identifies an agent that performs an action, the first verb encountered is the action being performed, and the next noun encountered is the recipient of the action (e.g., “The king [*agent*] assisted [*action*] the queen [*recipient of the action*]”). When sentences become longer, or the sentence meaning is represented with complex syntax, the cognitive challenge becomes greater (Just and Carpenter, 1992). The literature on sentence processing offers a number of reasons why this is so.

Early models of sentence comprehension postulated that, as a listener hears a sentence, the listener is continually forming hypotheses about the structure of what they are hearing and forming predictions about what they have yet to hear. These are working hypotheses, either confirmed or modified with the arrival of subsequent words of the sentence (cf., Frazier and Fodor, 1978; Fodor and Frazier, 1980; Marslen-Wilson and Tyler, 1980; Wanner, 1980). This general principle has been instantiated more recently in probability-based models of sentence processing that postulate that syntactically complex sentences are more difficult to understand because they violate the listener’s experience-based expectations of the likely structure of the sentence. This requires a re-analysis of the initially assumed structure, as, for example, that the first noun will be the agent of an action (cf., Novick et al., 2005; Levy, 2008; Padó et al., 2009; Gibson et al., 2013). In support of this view, whether a cause or consequence of the extra effort speakers and listeners must invest to produce and understand sentences with greater syntactic complexity, studies of everyday speech samples show that sentences with simpler syntactic forms occur far more frequently than sentences with more complex syntax (Goldman-Eisler, 1968; see also Goldman-Eisler and Cohen, 1970).

Consistent with the above observations, it is well known that, independent of hearing acuity, sentences with a variety of complex syntactic constructions are more difficult to comprehend and to recall than those with less complex structures, and that this is especially so for older adults (Feier and Gerstman, 1980; Emery, 1985; Kemper, 1986; Norman et al., 1991). Among the best-studied linguistic challenges in the literature are sentences that express their meaning with an object-relative syntactic structure versus sentences with a syntactically simpler subject-relative structure. Past studies have shown that not only do object-relative sentences produce more comprehension and recall errors than subject-relative sentences, but that this is differentially so for older than for younger adults (e.g., Carpenter et al., 1994; Wingfield et al., 2003). For this reason we have selected these two sentence types to form the basis for our analysis of a potential interaction between hearing acuity among older adults and the linguistic complexity of the speech materials.

The upper panel in **Table 1** shows an example of the simplest syntactic form we employed in the present study (base sentence): a six-word sentence with a subject-relative center-embedded clause structure, in which the main clause (*Sisters are fortunate*) is interrupted by a relative clause (*that assist brothers*). The more complex syntactic form we employed had an object-relative center-embedded clause structure. The first sentence in the lower set shows a sentence composed of the same six words, but now ordered such that the meaning is expressed with an object-relative construction. In this case the embedded clause (*that sisters assist*) not only interrupts the main clause, but the head noun phrase (*Brothers*) functions as both the subject of the main clause *(brothers are fortunate*) and the object of the relative clause (*that sisters assist*). Because the order of thematic roles in object-relative constructions is not canonical (the first noun is not the agent of the action), such sentences require a more extensive thematic integration than required for the more canonical structure represented by subject-relative sentences (Warren and Gibson, 2002). As a consequence, accurate comprehension of object-relative sentences has been considered to be more resource demanding than processing subject-relative sentences (e.g., Ferreira et al., 1996; Cooke et al., 2002).

**Table 1:** Examples of sentence types.

Sentence type		Distance between agent and action	Example sentence
Subject-relative	Six-words	Base sentence	Sisters that assist brothers are fortunate.
	10-words	Short separation	Sisters that assist brothers with short brown hair are fortunate.
	10-words	Long separation	Sisters with short brown hair that assist brothers are fortunate.
Object-relative	Six-words	Base sentence	Brothers that sisters assist are fortunate.
	10-words	Short separation	Brothers with short brown hair that sisters assist are fortunate.
	10-words	Long separation	Brothers that sisters with short brown hair assist are fortunate.


More specifically, it has been suggested that to determine the thematic roles in object-relative sentences one must keep the subject of the sentence in mind for a longer period of time than in subject-relative sentences (e.g., Cooke et al., 2002), which would be expected to place a heavier demand on working memory. Consistent with this likelihood have been studies showing that young and older adults with lower scores on tests of verbal working memory show more comprehension errors for complex sentences than those with better scores (e.g., Just and Carpenter, 1992; MacDonald et al., 1992; Carpenter et al., 1994; Vos et al., 2001). This working memory account has, either directly or indirectly, been used to account for the greater number of comprehension errors typically found for object-relative than for subject-relative sentences (Just and Carpenter, 1992; Zurif et al., 1995; Cooke et al., 2002; Wingfield et al., 2006), increased patterns of neural activation in functional imaging studies (Just et al., 1996; Cooke et al., 2002; Wingfield and Grossman, 2006; Peelle et al., 2010), and slower self-pacing patterns for both written (Stine-Morrow et al., 2000) and spoken (Waters and Caplan, 2001; Fallon et al., 2006) sentences.

### Increasing the Processing Challenge by Adding Prepositional Phrases

Although the non-canonical word order of object-relative sentences violates listeners’ experience-based expectancies, a major source of the above-cited difficulty with such sentences, as argued by, for example, Cooke et al. (2002) and Warren and Gibson (2002), is a word-order that impedes successful semantic integration of the lexical elements in the sentence. If this were the case, then whether a sentence has a subject-relative or an object-relative structure, a manipulation that further increases the difficulty of the semantic integration of the sentence elements would be expected to increase failures of correct comprehension of the sentence meaning. Central to our primary interest, however, is the question of whether one would see an exaggeration of any effects of this added degree of linguistic challenge on sentence comprehension in listeners with reduced hearing acuity.

To test these hypotheses, 10-word sentences were created from the six-word base sentences by inserting a four-word prepositional phrase (e.g., *with short brown hair*) into each of the six-word base sentences. Moreover, the particular placement of the prepositional phrase manipulated the processing challenge by manipulating the separation between key sentence constituents. In a less syntactically disrupting case the placement of the prepositional phrase kept the person performing the action and the action being performed adjacent to, or in close proximity with, each other. These are indicated in **Table 1** as *short separation* sentences. The second sentence in the upper set illustrates such a propositional phrase placement for a subject-relative sentence (*short separation*). The second sentence in the lower set shows this for an object relative sentence. (In the table, we have underlined the agent performing the action and the action being performed.)

In the second type of placement the prepositional phrase was inserted in a position to produce a long separation between the person performing the action and the action being performed. This placement was designed to add difficulty to the task of determining the thematic role assignments of the two persons in the sentence, and a presumed increase in working memory demands, but without changing the formal syntactic structure of the sentence itself. Examples of such *long separation* sentences are shown in **Table 1** for a subject-relative sentence (upper set) and an object-relative sentence (lower set). If object-relative sentences prove more difficult, this manipulation would allow us to dissociate the challenging grammatical features of this sentence from the increased difficulty associated with the increased separation between the key sentence constituents.

By having sentences in which males (e.g., brother) or females (e.g., sister) as the agents or recipients of actions, accurate comprehension could be demonstrated by the participant correctly indicating the gender of the agent of the action. (In these examples and in the experiment itself the complementizer *that* was used instead of the more grammatically correct *who.* This was done to avoid the use of the *who*–*whom* distinction that could serve as an undesired comprehension cue.)

The target groups in this experiment were older adults with good hearing acuity and an age-matched group with a mild-to-moderate hearing loss. A third group of participants consisting of young adults with normal hearing acuity was included to illustrate the maximal performance level that might be expected under ideal circumstances.

### Presentation Level

Hearing research over the years has reflected a choice among the intensity levels that might be used: whether to present speech at an intensity that approximates conversational speech levels (dB HL or SPL; Hearing Level or Sound Pressure Level) or at a presentation level relative to an individual’s hearing threshold (dB SL; Sensation Level). Experimental studies typically employ either one presentation method or the other; rarely both within the same experiment. This leaves open the question of whether the two methods will be equally sensitive to the factors of interest in a particular study. For this reason in the present study, we employed both of the presentation methods (the same absolute presentation level for all participants [dB HL] and a presentation level adjusted for each individual’s hearing threshold [dB SL]) using a within-participants design. Including both sound presentation levels would thus allow us to see whether both methods may reveal an influence on the factors tested in this sentence processing task equally, and to provide useful empirical information in helping to determine which approach may be more appropriate in future studies. Thus, uniquely within a single experiment, we manipulate syntactic complexity, the effect of a separation of key sentence elements by insertion of a prepositional phrase, and presentation level of the sentence stimuli within the context of adult aging and hearing acuity.

### Experimental Hypotheses

One could entertain two hypotheses in terms of sentence comprehension in older adults with good or poor hearing acuity. The first is that perceptual effort – as determined by participants’ hearing acuity and presentation level – will have similar effects on sentence comprehension regardless of the cognitive load imposed by syntactic complexity, sentence length, and prepositional phrase placement. This simple additivity would be manifested in parallel comprehension performance functions for the good-hearing and hearing-impaired listeners, albeit with a potential difference in y-intercepts. A finding of additivity would be consistent with the notion of independence of cognitive and perceptual operations. (See Allport et al., 1972 and McLeod, 1977, for early arguments favoring multiprocessor models of attention.)

The alternative would be a multiplicative effect, in which perceptual effort engendered by reduced hearing acuity and/or reduced presentation amplitude, produces a differentially greater negative effect on comprehension of the more cognitively challenging sentences (object-relative sentences with a long agent-action separation) than on comprehension of the less challenging sentences (subject-relative sentences with a short agent-action separation).

This latter finding would be in keeping with the principles embodied in models that postulate limited attentional (Kahneman, 1973; Cowan, 1999; Engle, 2002) or working memory (Baddeley, 2012; Chow and Conway, 2015) resources that must be shared among concurrent or closely sequential processing operations. Applied to the present case, this would imply that the resources required for front-end perceptual operations will necessarily draw on the resources that would otherwise be available for comprehension operations at the linguistic processing level. Such an effect would thus predict that the consequences of the extra resource draw necessary for successful perceptual processing of an acoustically degraded speech input will fall more heavily on successful comprehension of the more resource-demanding long separation object relative sentences than their less syntactically challenging counterparts.

## Materials and Methods

### Participants

Participants were 36 older adults, 18 with good hearing acuity (5 males and 13 females) and 18 older adults with a mild-to-moderate hearing loss (6 males and 12 females). Audiometric assessment was conducted using a GSI 61 clinical audiometer (Grason-Stadler, Madison, WI, USA) using standard audiometric procedures in a sound attenuating testing room.

**FIGURE 1:**
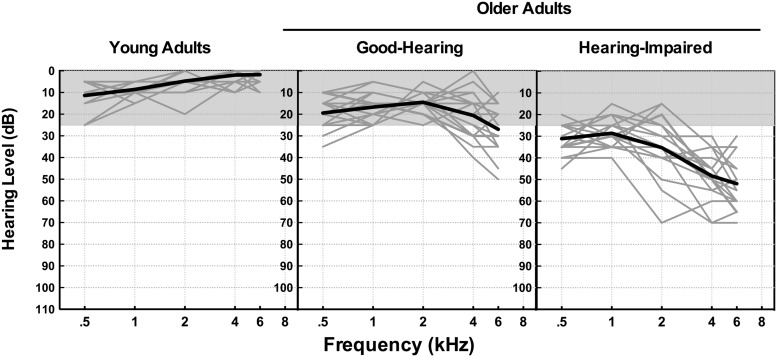
**Better-ear pure-tone thresholds from 0.5 kHz to 8 kHz for the three participant groups.** Hearing profiles for individual listeners within each participant group are shown in light gray, with the group average drawn in black. The shaded area in each of the panels indicates thresholds less than 25 dB HL.

**Figure 1** shows better-ear pure-tone thresholds from 500 to 8,000 Hz for the three participant groups plotted in the form of audiograms, with the *x*-axis showing the test frequencies and the *y*-axis showing the minimum sound level (dB HL) needed for their detection. Hearing profiles for individual listeners within each participant group are shown in light gray, with the group average drawn in black. The shaded area in each of the panels indicates thresholds less than 25 dB HL, a region commonly considered as clinically normal hearing for speech (Katz, 2002).

We summarized individuals’ hearing acuity in terms of their better-ear pure tone average (PTA) across.5, 1, 2, and 4 kHz, a range especially important for the perception of speech. The participants in the older adult good-hearing group had a mean PTA of 17.9 dB HL (*SD* = 3.0). The older adult hearing-impaired group had a mean PTA of 35.8 dB HL (*SD* = 5.7) placing them in the mild-to-moderate hearing loss range (Katz, 2002). This degree of loss represents the single largest proportion of hearing-impaired older adults (Morrell et al., 1996). None of the participants in the present study reported regular use of hearing aids, and all were tested unaided.

The good-hearing and hearing-impaired older adult groups were similar in age, with the good-hearing group ranging from 68 to 84 years (*M* = 74.0 years, *SD* = 4.6) and the hearing-impaired group ranging from 67 to 83 years (*M* = 74.5 years, *SD* = 5.2; *t*[34] = 0.31, *n.s*.). Both groups were well educated, with a mean of 16.5 years of formal education for the good-hearing group (*SD* = 2.2) and 17.2 years for the hearing-impaired group (*SD* = 2.5); *t*(34) = 0.84, *n.s*.. The two groups were also similar in vocabulary knowledge as measured by a 20-item version of the Shipley vocabulary test (Zachary, 1991). This is a written multiple choice test in which the participant is required to indicate which of four listed words means the same or nearly the same as a given target word. The good-hearing older adults had a mean score of 17.3 (*SD* = 2.4) and the hearing-impaired older adults had a mean score of 17.4 (*SD* = 2.4); *t*(34) = 0.14, *n.s*..

For purposes of comparison we also included a group of 18 younger adults (three males, 15 females), ranging in age from 18 to 29 years (*M* = 20.4; *SD* = 2.7), all of whom had age-normal hearing acuity, with a mean PTA of 6.7 dB HL (*SD* = 3.1). At time of testing the young adults had completed fewer years of formal education (*M* = 14.6 years; *SD* = 1.1) than either the good-hearing, *t*(34) = 3.29, *p* < 0.01, or the hearing-impaired, *t*(34) = 4.02, *p* < 0.001, older adults. As is common in adult aging (e.g., Verhaeghen, 2003), the young adults had somewhat lower vocabulary scores (*M* = 13.9; *SD* = 2.3) than either the good-hearing, *t*(34) = 4.41, *p* < 0.001, or the hearing-impaired, *t*(34) = 4.47; *p* < 0.001, older adults.

All participants reported themselves to be in good health, with no history of stroke, Parkinson’s disease, or other neuropathology that might compromise their ability to carry out the experimental task. All participants reported themselves to be monolingual native speakers of American English with no history of speech or language disorders.

### Working Memory Capacity

Although varying in emphasis, the term working memory has been typically used to refer to the retention of information in conscious awareness when this information is not present in the environment, to its manipulation, and to its use in guiding behavior (Postle, 2006; see also McCabe et al., 2010; Baddeley, 2012, for converging definitions). In accord with this definition, tests of working memory typically focus on complex span tasks in which material must be held in memory while other operations, either related or unrelated to the material in memory, must be performed (Baddeley and Hitch, 1974). A common assessment of verbal working memory that meets this definition is the reading span task introduced by Daneman and Carpenter (1980), and its variants (e.g., Baddeley et al., 1985; Waters and Caplan, 1996; Conway et al., 2005; Moradi et al., 2014).

For all participants working memory capacity was assessed using the reading span task modified from Daneman and Carpenter (1980; Stine and Hindman, 1994). In this task participants read sets of sentences and responded after each sentence whether the statement in the sentence was true or false. Once a full set of sentences had been presented participants were instructed to recall the last word of each of the sentences in the order in which the sentences had been presented. The task thus requires the participant to make a true-false decision about the statement in each sentence while simultaneously holding the final words of each of the prior sentences in memory. McCabe et al.’s (2010) stair-step presentation was used, in which participants received three trials for any given number of sentences, with a working memory score calculated as the total number of trials in which all sentence-final words were recalled correctly in the correct order. The maximum score on this test is 15.

The reading span task was chosen because it draws heavily on both storage and processing components of working memory (Daneman and Carpenter, 1980), and in written form would not be confounded with hearing acuity. As illustrated in, for example, a meta-analysis of published studies reported by Daneman and Merikle (1996), reading span scores have been shown to be a good predictor of performance in a variety of language processing tasks.

**FIGURE 2:**
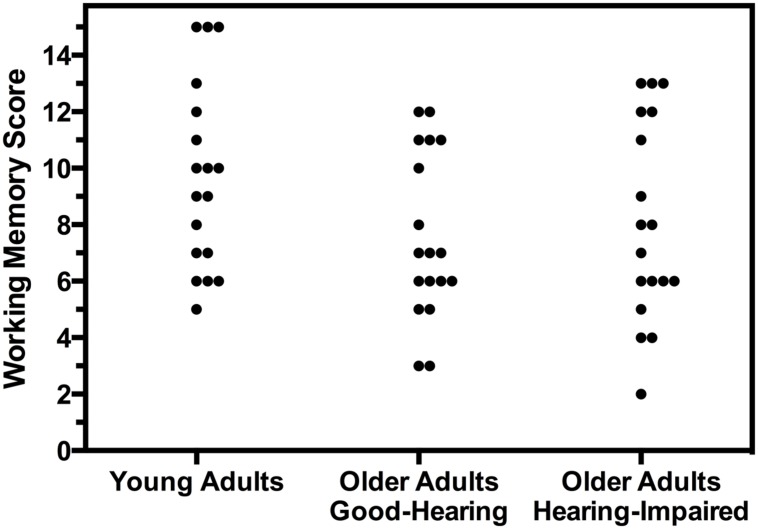
**Individual reading span scores as a measure of working memory capacity.** Scores are shown separately for young adults with age-normal hearing acuity (*young adults*), older adults with clinically normal hearing acuity for speech (*good-hearing*) and older adults with mild-to-moderate hearing loss (*hearing-impaired*).

**Figure 2** shows a plot of the working memory (reading span) scores for each of the young adults and each of the good-hearing and hearing-impaired older adults taking part in the experiment. The variability within groups and the overlap between groups stands out clearly. Given this variability there was no significant difference between the scores for two older adult groups, *t*(34) = 0.46, *n.s*.. Although **Figure 2** shows a tendency for the young adults’ distribution to be shifted higher relative to the two older adult groups, the overall difference showed only a non-significant trend as compared with the good-hearing older adults, *t*(34) = 2.03, *p* = 0.051, and no significant difference relative to the hearing-impaired older adults, *t*(34) = 1.42, *p* = 0.17.

### Stimuli

Preparation of the stimuli began with construction of 144 six-word English sentences with a subject-relative structure. In each sentence a male agent (e.g., boy, uncle, king) or a female agent (e.g., girl, aunt, queen) was performing an action (e.g., pushed, helped, teased). In half of the sentences the male was the agent of the action and in half the female was the agent. For each of these sentences a counterpart sentence was then constructed using the same words but with the meaning expressed with an object-relative structure. In addition, for each of these subject-relative and object-relative sentences a plausible four-word prepositional phrase was inserted in a position that kept at most a one word separation between the person performing the action and the action being performed (*short separation*) or placed so as to separate the person performing the action and the action being performed by at least four intervening words (*long separation*). Examples of these six sentence types are illustrated in the previously described **Table 1.**

The resulting 864 sentences were recorded by a female speaker of American English to form the master stimulus set. Sentences were recorded with natural intonation at an average speaking rate of 150 words per minute onto sound files using Sound Studio v2.2.4 (Macromedia, Inc., San Francisco, CA, USA) that digitized (16-bit) at a sampling rate of 44.1 kHz. Recordings were equalized within and across sentence types for root-mean-square (RMS) intensity using MATLAB (MathWorks, Natick, MA, USA). There were also 48 filler sentences prepared that consisted of six- to nine-word active-conjoined sentences that were similar in content to the test sentences but that did not contain an embedded clause structure.

### Procedures

Each participant heard 144 test sentences, 24 in each of the six sentence types (six-word subject-relative, six-word object-relative, 10-word subject-relative short separation, 10-word subject-relative long separation, 10-word object-relative short separation, 10-word object-relative long separation) along with the 48 filler sentences. Participants were instructed to listen to each sentence as it was presented and then to indicate whether it was either the male or the female in the sentence that was performing the action. Responses were made by pressing the correct one of two keys labeled male or female.

Half of the sentences of each type (six-word subject-relative, six-word object-relative, 10-word subject-relative short separation, 10-word subject-relative long separation, 10-word object-relative short separation, 10-word object-relative long separation) were presented to participants at 65 dB HL, a level that approximates everyday conversational speech. The remaining half of each sentence type was presented at 20 dB above each the participant’s better-ear PTA (i.e., 20 dB SL; Jerger and Hayes, 1977). Stimuli were presented binaurally over Eartone 3A insert earphones (E-A-R Auditory Systems; Aero Company, Indianapolis, IN, USA) via a Grason Stadler GS-61 clinical audiometer (Grason-Stadler, Inc., Madison, WI, USA) in the same sound-isolated testing room in which hearing acuity was tested.

A within-participants design was used in which each participant received equal numbers of sentences of each type, with no base sentence (a particular combination of agent, recipient and action) heard more than once by any participant. Sentences and sound level presentation conditions were counterbalanced across participants such that, by the end of the experiment, each base sentence had been heard an equal number of times in each of its syntactic and agent-action separation versions and at 65 dB HL and 20 dB SL an equal number of times. Sound levels were blocked in presentation, with the order of sound level blocks counterbalanced across participants. Sentence types were randomized in order of presentation within the sound-level blocks. Written informed consent was obtained from all participants according to a protocol approved by the Brandeis University Institutional Review Board prior to the start of the experiment.

### Audibility Testing

To insure audibility of the stimuli participants were presented with two sentences at 65 dB HL, and two sentences at 20 dB SL for that individual, with one sentence at each intensity level having a subject-relative structure and one with an object-relative structure. The participant’s task was simply to repeat each sentence aloud as it was heard. None of these sentences was used in the main experiment. The good-hearing older adults had 100% word report accuracy at both 65 dB HL and 20 dB SL. The older adult hearing-impaired group scored a mean of 99.5% correct at 65 dB HL and 100% correct at 20 dB SL. The young adults scored 100% correct at 65 dB HL and 99.8% correct at 20 dB SL.

## Results

**FIGURE 3:**
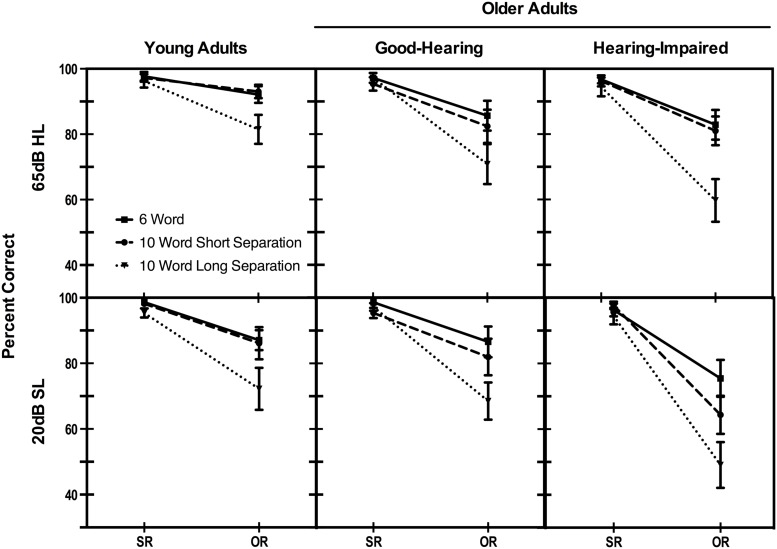
**Comprehension accuracy for six-word sentences with a subject-relative (SR) or object-relative (OR) structure and 10-word subject-relative and object-relative sentences with a prepositional phrase positioned to produce either a short or long separation between the agent and action represented in the sentence.** Data are shown for three participant groups hearing sentences at a uniform 65 dB Hearing Level (HL) for all participants or at 20 dB Sensation Level (SL) relative to individuals’ auditory thresholds. Error bars represent 1 SE.

The main results are summarized in **Figure 3** that shows the percentage of correct comprehension responses for subject-relative and object-relative six-word, 10-word short separation and 10-word long separation sentences when heard at 65 dB HL and at 20 dB SL for the three participant groups. Consistent with expectations it can be seen that comprehension of sentences with the syntactically simpler subject-relative structures was excellent for all three participant groups regardless of sound-level condition, sentence length, or prepositional phrase placement. The ceiling and near-ceiling level performance for the subject relative sentences also confirms the basic audibility of sentences heard with both sound-level presentations. Differences in comprehension accuracy begin to appear, however, when the syntactic complexity of the sentences was increased by expressing the meaning with an object-relative structure.

An omnibus mixed design analysis of variance (ANOVA) was conducted on the comprehension accuracy data shown in **Figure 3** that included effects of syntactic structure (2: subject-relative, object-relative), length manipulation (3: six-word sentences, 10-word short subject-action separation, 10-word long subject-action separation), participant group (3: young adults, older good-hearing, older hearing-impaired) and presentation level (2: 65 dB HL, 20 dB SL). Participant group was a between-participants variable; all others were within-participants variables. Because of ceiling effects constraining variance for the subject-relative sentences, we performed all ANOVAs and paired-comparison *t*-tests on rationalized arcsine transformed data (Studebaker, 1985).

The ANOVA confirmed a significant main effect of syntactic structure, reflecting the previously cited common finding of poorer comprehension accuracy for the more computationally demanding object-relative sentences than for the less demanding subject-relative sentences, *F*(1,51) = 106.09, *p* < 0.001, $\eta_{p}^{2}$ = 0.68. Although this effect of complex syntax on comprehension accuracy held across all three participant groups, the relative size of the effect differed between participant groups as reflected in a significant Syntactic structure × Participant group interaction, *F*(2,51) = 4.00, *p* < 0.05, $\eta_{p}^{2}$ = 0.14.

Of greater interest, the ANOVA also confirmed a main effect of the sentence length manipulation, *F*(2,102) = 39.32, *p* < 0.001, $\eta_{p}^{2}$ = 0.44. As can be seen from visual inspection of **Figure 3**, however, this main effect was moderated by a significant Length × Syntactic structure interaction, *F*(2,102) = 24.11, *p* < 0.001, $\eta_{p}^{2}$ = 0.32, confirming that the effect of length had its effect only for the more syntactically complex object-relative sentences. There was also a significant main effect of participant group, *F*(2,51) = 3.24, *p* < 0.05, $\eta_{p}^{2}$ = 0.11.

Although both presentation sound levels were suprathreshold, as confirmed by the previously cited audibility check, the uniform presentation level of 65 dB HL was relatively louder than the 20 dB SL presentation level for all three participant groups. This difference resulted in a significant main effect of presentation level on comprehension accuracy, *F*(1,51) = 9.03, *p* < 0.01, $\eta_{p}^{2}$ = 0.15. Because the 20 dB SL presentation levels were based on individuals’ pure tone thresholds, the size of the difference between these values and the 65 dB uniform presentation level was inversely proportional to participants‘ baseline hearing acuity. The relative effect of the sound level, however, did not differ by group, as seen in the lack of a significant Presentation level × Participant group interaction, *F*(2,51) = 1.53, *p* = 0.23, $\eta_{p}^{2}$ = 0.06. The effect of presentation level, however, had a greater effect on comprehension accuracy for the object-relative sentences than for the subject-relative sentences, with comprehension accuracy for subject-relative sentences at ceiling or near ceiling for both presentation levels, resulting in a significant Presentation level × Syntactic structure interaction, *F*(1,51) = 12.53, *p* = 0.001, $\eta_{p}^{2}$ = 0.20. None of the remaining interactions was significant.

We conducted a series of follow-up ANOVAs and paired-comparisons to explore in more detail the factors underlying this pattern of main effects and interactions. Because comprehension accuracy for subject-relative sentences was at or near ceiling for all participants and all conditions, the analyses that follow were conducted on the data for just the object-relative sentences.

For the young adults a two-way ANOVA conducted on comprehension accuracy showed a significant main effect of sentence length (*p* < 0.001) and of presentation level (*p* < 0.05**),** but no Length × Presentation level interaction (*p* = 0.65). Follow-up paired comparison testing failed to show a significant difference between the six-word sentences and the 10-word short separation sentences for either the 65dB HL (*p* = 0.99) or the 20 dB SL (*p* = 0.15) presentation levels. That is, the significant effect of sentence length was due to the poorer comprehension for the 10-word long separation sentences relative to the six-word sentences and the 10-word short separation sentences for both presentations levels (*p* levels < 0.05 to < 0.01). The difference in comprehension accuracy for the two presentation levels failed to reach significance for either the six-word sentences (*p* = 0.12) or for the 10-word short separation sentences (*p* = 0.43). There was a non-significant trend toward an effect of presentation level for the 10-word long separation sentences (*p* = 0.053).

For the good-hearing older adults a two-way ANOVA conducted on comprehension accuracy showed a significant main effect of sentence length (*p* < 0.001) but neither a significant main effect of presentation level (*p* = 0.45), nor a Length × Presentation level interaction (*p* = 0.53). Similar to the data for the young adults, paired-comparison testing failed to show a significant difference between the six-word sentences and the 10-word short separation sentences for either the 65dB HL (*p* = 0.15) or the 20 dB SL (*p* = 0.15) presentation levels. As with the young adults there was again poorer comprehension for the 10-word long separation sentences relative to the six-word sentences and 10-word short separation sentences for both presentations levels (*p* levels < 0.01 to < 0.001). The difference in comprehension accuracy for the two presentation levels failed to reach significance for either the six-word sentences (*p* = 0.94), the 10-word short separation sentences (*p* = 0.76), or the 10-word long separation sentences (*p* = 0.20).

For the hearing-impaired older adults several of the trends seen for the better-hearing groups were now more marked. A two-way ANOVA conducted on comprehension accuracy for the hearing-impaired participants showed significant main effects of sentence length (*p* < 0.001) and presentation level (*p* = 0.001). There was no Length × Presentation level interaction (*p* = 0.51). Although the ANOVA failed to yield a significant Length × Presentation level interaction, planned comparison tests showed no significant difference between the six-word sentences and 10-word short separation sentences at 65dB HL (*p* = 0.36) but this difference did reach significance with the more challenging 20 dB SL presentation (*p* < 0.01). The 10-word long separation sentences showed significantly poorer comprehension accuracy than both the six-word and 10-word short separation sentences at both presentation levels (*p* levels < 0.01 to < 0.001). The difference in comprehension accuracy for the two presentation levels was significant for the six-word sentences (*p* < 0.05), the 10-word short separation sentences (*p* < 0.01), and the 10-word long separation sentences (*p* < 0.01).

A final analysis was conducted to compare the two target groups with each other: the good-hearing older adults versus the hearing-impaired older adults. The two groups’ comprehension accuracy was similar for the six-word sentences at the 65 dB HL (*p* = 0.60), with a trend toward a difference emerging at the 20 dB SL presentation level (*p* = 0.054). A developing pattern was seen for the 10-word short separation sentences which failed to show a significant difference between the two groups at 65 dB HL (*p* = 0.64), but a significant difference between the two participant groups did appear for the 20 dB HL presentation level (*p* < 0.05). For the 10-word long separation sentences there was again no significant difference between groups at 65 dB HL (*p* = 0.27) but there was a small but significant difference between groups at 20 dB SL (*p* < 0.05), potentially constrained by the previously noted functional floor of chance level performance for the 10-word long separation sentences with a 20 dB SL presentation level.

### Effects of Working Memory, Hearing Acuity, and Age as Continuous Variables

Although the good-hearing and hearing-impaired older adults were equivalent in mean age and reading span scores, there was, as seen, within-group variability in age, reading span, and hearing acuity. The error bars seen in **Figure 3** also indicate some variability around the plotted means. To explore the factors that may have led to the variability in comprehension accuracy we carried out hierarchical multiple regressions separately for the two presentation levels, first to see what factors may have contributed to comprehension performance and second, to determine whether the pattern of relative contributions generalized across presentation levels. In these analyses we considered just the older adults rather than including the young adults to avoid the multiple differences between the young and older adult groups potentially biasing the regression outcomes.

The dependent variable in each case was comprehension accuracy for the object-relative sentences due to the ceiling and near-ceiling performance for both participant groups for comprehension of the subject-relative sentences in all three length conditions and the two sound-level conditions. Predictor variables were entered into the model in the following order: working memory span (represented by reading span score), hearing acuity (represented by the better ear PTA, averaged over 500, 1000, 2000, and 4000 Hz), and participants’ chronological age in years. This order was selected to examine any contribution of hearing acuity beyond effects of working memory span, and to determine whether age contributed unique variance after accounting for working memory span and hearing acuity.

The results of the regression analyses are shown in **Table 2.** For each predictor variable in each of the two presentation level conditions we show *R*^2^, which represents the cumulative contribution of each variable along with the previously entered variables, and the change in *R*^2^, which shows the contribution of each variable at each step. The next column shows the level of significance of each variable and the final column shows the standardized regression coefficients (β). It can be seen that working memory as measured by reading span is a significant predictor of comprehension accuracy for all conditions in the experiment; for both the six- and 10-word sentences and in the latter case for the short and long agent-action separations for both the 65 dB HL and the 20 dB SL presentation levels.

**Table 2:** Hierarchical regression analyses for object-relative sentences.

		Predictor	*R*^2^	Change in *R*^2^	*p*^∗^	β^†^
65 dB HL	Six-word	Reading span	0.09	0.09	0.07	0.26
		Hearing acuity	0.10	0.01	0.61	-0.06
		Age	0.13	0.03	0.33	-0.17
	10-word short separation	Reading span	0.13	0.13	0.05	0.32
		Hearing acuity	0.14	0.01	0.50	-0.10
		Age	0.15	0.01	0.54	-0.11
	10-word long separation	Reading span	0.32	0.32	0.001	0.56
		Hearing acuity	0.39	0.07	0.06	-0.26
		Age	0.39	0.00	0.99	0.00
20 dB SL	Six-word	Reading span	0.25	0.25	0.01	0.43
		Hearing acuity	0.31	0.07	0.09	-0.22
		Age	0.36	0.05	0.14	-0.23
	10-word short separation	Reading span	0.17	0.17	0.025	0.37
		Hearing acuity	0.31	0.15	0.025	-0.37
		Age	0.32	0.01	0.60	-0.08
	10-word long separation	Reading span	0.20	0.18	0.01	0.38
		Hearing acuity	0.33	0.15	0.025	-0.35
		Age	0.36	0.03	0.26	-0.17


*^∗^*p-*value reflects significance of change in *R*^*2*^ at each step of the model.*

*^†^Standardized multiple regression co-efficient.*

When the presentation level was at the higher 65 dB HL level, hearing acuity contributed to comprehension accuracy only for the 10-word sentences with a long agent-action separation. When the perceptual task was more challenging in the 20 dB SL condition hearing acuity contributed marginally for the six-word sentences, increasing to a significant contribution for the 10-word short and long separation sentences. That is, hearing acuity contributed significant variance only for the more challenging presentation level and even then only for the longer 10-word sentences. With the contributions of working memory span and hearing acuity taken into account, chronological age did not contribute additional variance to comprehension accuracy. (The same pattern as shown in **Table 2** also appeared when the data for the young adults were included in the regression analyses.)

## Discussion

Although hearing loss is a common accompaniment of adult aging, it has primarily been considered as an independent issue in aging research. There is now a growing recognition, however, that successful speech comprehension reflects an adaptive interaction between sensory and cognitive operations. There are two aspects to this interaction. The first is that the poorer the acoustic quality of the stimulus, whether due to reduced hearing acuity, poorly articulated speech, or the presence of background noise, the more support is required from top-down linguistic knowledge (Lindblom et al., 1992; Wingfield et al., 1994; Pichora-Fuller, 2003; Benichov et al., 2012; Rönnberg et al., 2013). In the present experiment this successful balance was revealed in the excellent level of comprehension success for six- and 10-word meaningful sentences by both good-hearing and hearing-impaired older adults at both presentation levels so long as the sentence meanings were expressed with the syntactically less complex subject-relative construction.

It is the case that all participants, to include those in the older adult hearing-impaired group, successfully scored at ceiling or near ceiling when tested for speech audibility at both sound intensity levels we employed. This should not imply, however, that all groups had access to the same quality of stimulus input. This is the other side of the sensory–cognitive interaction; namely, the previously cited position that successful perception in the face of an acoustically degraded stimulus may come at the cost of resources that would otherwise be available for higher-level cognitive or linguistic operations. This position, in its broad outlines, has sometimes been referred to as an “effortfulness hypothesis” (Rabbitt, 1968, 1991; see also Surprenant, 1999, 2007; Murphy et al., 2000; Pichora-Fuller, 2003; McCoy et al., 2005; Wingfield et al., 2005, 2006; Amichetti et al., 2013, for similar arguments).

So long as the processing demands required for sentence comprehension did not exceed an upper limit on total processing resources, as in the case of sentences with a subject-relative structure, successful comprehension was possible even under conditions of perceptual effort. According to this resource argument, this point would have been exceeded when the difficulty in determining the thematic role assignments within a sentence imposed additional processing demands beyond those required for resolution of subject-relative sentences and when greater listening effort was required. This effect was revealed in reduced accuracy for object-relative sentences and when the relational elements were separated by insertion of a prepositional phrase in the long agent-action separation condition. This latter placement would be expected to exacerbate the already greater difficulty in determining thematic roles in object-relative constructions as the relational elements would need to be held in memory for a longer period of time (see Cooke et al., 2002, for a similar argument). The pattern of contributions of working memory and hearing acuity across conditions in the regression analyses is consistent with this argument. It is interesting that, at least for these data, chronological age contributed little to the variance in comprehension accuracy once working memory and hearing acuity were taken into account.

The effortfulness hypothesis, which is consistent with extant models that postulate an upper limit on working memory or attentional resources (cf., Kahneman, 1973; Baddeley and Hitch, 1974), has some descriptive utility as an account for our central question of why reduced hearing acuity results in a differentially greater effect on comprehension of object-relative than on subject-relative sentences even though all sentences were presented at a supra-threshold level that insured audibility of the recorded stimuli.

An additional factor that may be considered can be referred to as an expectancy-uncertainty based account. As noted previously, because object-relative and other syntactically complex forms occur less frequently in one’s everyday listening experience than simpler syntactic forms (e.g., Goldman-Eisler, 1968; Goldman-Eisler and Cohen, 1970), one’s expectations of encountering such forms would consequently be lower. In an early formulation Osgood (1963) focused on expectations at the form-class level; the likelihood, for example, that a noun phrase will be followed by a verb, and a verb will be followed by a noun phrase. Later formulations have combined both syntactic and semantic elements to account for the greater difficulty listeners are known to have for sentences that express their meaning with complex syntax. This is the postulate that the listener’s experience-based expectation that the first noun will be the agent of an action will have to be rejected as a sentence with an object-relative construction unfolds and this expectation is disconfirmed. Elements of this postulate can be seen in a number of expectancy inclusive models of sentence comprehension (cf., Hale, 2001; Novick et al., 2005; Levy, 2008; Padó et al., 2009; Gibson et al., 2013).

It should be noted in this discussion that we do not present working memory and experience-based expectation accounts as mutually exclusive alternatives. Indeed, a study examining eye-movements in reading text has implicated contributions to sentence processing from both sources (Staub, 2010).

Although an expectancy-based account might apply to the traditional finding of greater comprehension errors for object-relative sentences, it would not, in itself, explain why reduced hearing acuity would exacerbate this effect. An expectancy-based account, however, must not only include the likelihood of encountering a particular lexical item or structural form. It must also include an element of uncertainty, sometimes referred to as response entropy (see Shannon and Weaver, 1949). Here this would be represented by the number and probability strengths of alternative perceptual interpretations of the acoustic signals representing relationally critical words in the sentences. Studies of word recognition from reduced acoustic information have shown that alternative possibilities fitting an ambiguous acoustic signal may be activated by sentential context (Lash et al., 2013) and phonological similarity with other words Sommers, 1996). Activation of a wider array of lexical possibilities might be expected to arise when the acoustic specificity of a word is reduced, as would be the case with poor hearing acuity, compounded by the lower presentation level in the 20 dB SL condition. Support for the influence of both expectation and entropy in spoken word recognition can be seen in studies of words presented in noise or with reduced word onset information, with the uncertainty (entropy) effect stronger for older than for younger adults (cf., van Rooij and Plomp, 1991; Lash et al., 2013).

### Limitations of the Present Study

First, it is important to note that the participants in this experiment represented high-functioning older adults with good verbal knowledge and working memory capacity. Indeed, as a group, the good-hearing and hearing-impaired older adults had better vocabulary scores than their younger adult counterparts and a distribution of working memory span scores that were relatively close to that of the young adults. It should also be emphasized that stimuli were presented in quiet, thus avoiding the special difficulty older adults have when hearing speech with background noise (Humes, 1996; Tun et al., 2002). With less cognitively able older adults and/or with speech heard in noise one might expect even greater effects of age, hearing acuity, and working memory capacity on comprehension accuracy. As reviewed by Mattys et al. (2012), these variables do not exhaust the potential adverse conditions that might affect speech comprehension, to include accented speech and listening while engaging in a concurrent secondary activity.

Second, although we have made reference to listening effort, it must be acknowledged that both its definition and measurement remain a topic of debate (McGarrigle et al., 2014). It should also be acknowledged that definitions of working memory and its relation to attentional resources and executive function remain in contention (cf., Cowan, 1999, 2005; Miyake et al., 2000; Engle, 2002; Barrouillet et al., 2004; McCabe et al., 2010; Baddeley, 2012; Chow and Conway, 2015). It is possible that differences in tasks and in task demands may tap different components of a complex working memory system (cf., Akeroyd, 2008; Schoof and Rosen, 2014; Füllgrabe et al., 2015). Finally, specific to the psycholinguistics literature, there is also the question of whether language comprehension is carried by a specialized or more general working memory system (Wingfield et al., 1998; Caplan and Waters, 1999).

## Conclusion

Declines in sensory acuity and efficiency of cognitive function often co-occur in adult aging. Both can affect speech comprehension, with the interaction between the two revealed in the dual challenges of hearing impairment and syntactic complexity in determination of semantic relations in sentence comprehension. It should also be noted that although our focus has been on downstream effects of listening effort, deficits in recall and comprehension of written text with degraded vision have also been reported in the literature (Dickinson and Rabbitt, 1991; Gao et al., 2012). This suggests that the principles of sensory–cognitive interactions under study in this present paper have wider application to issues in adult aging even beyond hearing acuity and listening effort.

## Author Contributions

All authors listed, have made substantial, direct and intellectual contribution to the work, and approved it for publication.

## Conflict of Interest Statement

The authors declare that the research was conducted in the absence of any commercial or financial relationships that could be construed as a potential conflict of interest.
